# Supplementary material for: A fault-intrusion-tolerant system and deadline-aware algorithm for scheduling scientific workflow in the cloud
Source: PeerJ Comput Sci. 2021 Nov 2;7:e747. doi: 10.7717/peerj-cs.747 (PMC8576554; doi:10.7717/peerj-cs.747)
Supplement: Supplemental Information 6 [file peerj-cs-07-747-s006.zip › Myproject1.1/docs/index-files/index-9.html]

I-Index


JavaScript is disabled on your browser.


- Overview
- Package
- Class
- Use
- Tree
- Deprecated
- Index
- Help

- Prev Letter
- Next Letter

- Frames
- No Frames

- All Classes

A B C D E F G H I J L M N O P Q R S T U V W Z 


## I

id - Variable in class org.cloudbus.cloudsim.network.datacenter.Switch


ImpactFactorVariance - Class in org.workflowsim.clustering.balancing.metrics
:   ImpactFactorVariance is the standard deviation of their impact factors

ImpactFactorVariance() - Constructor for class org.workflowsim.clustering.balancing.metrics.ImpactFactorVariance


incrementMigrationCount() - Method in class org.cloudbus.cloudsim.power.PowerDatacenter
:   Increment migration count.

incrementVmsAcks() - Method in class org.cloudbus.cloudsim.DatacenterBroker
:   Increment vms acks.

incrementVmsAcks() - Method in class org.cloudbus.cloudsim.network.datacenter.NetDatacenterBroker
:   Increment vms acks.

index2job - Static variable in class org.workflowsim.failure.FailureMonitor
:   Id to a Job.

indexOf(List<T>, int, int) - Static method in class org.cloudbus.cloudsim.lists.ResCloudletList
:   Finds the index of a Cloudlet inside the list.

INEXEC - Static variable in class org.cloudbus.cloudsim.Cloudlet
:   The Cloudlet is in execution in a Cloud node.

InfoPacket - Class in org.cloudbus.cloudsim
:   InfoPacket class can be used to gather information from the network layer.

InfoPacket(String, int, long, int, int, int) - Constructor for class org.cloudbus.cloudsim.InfoPacket
:   Constructs a new Information packet.

INFOPKT\_RETURN - Static variable in class org.cloudbus.cloudsim.core.CloudSimTags
:   This tag is used to return the ping request back to sender

INFOPKT\_SUBMIT - Static variable in class org.cloudbus.cloudsim.core.CloudSimTags
:   This tag is used by an entity to send ping requests

init(int, Calendar, boolean) - Static method in class org.cloudbus.cloudsim.core.CloudSim
:   Initialises CloudSim parameters.

init(int, Calendar, boolean, double) - Static method in class org.cloudbus.cloudsim.core.CloudSim
:   Initialises CloudSim parameters.

init(String) - Method in class org.cloudbus.cloudsim.examples.power.planetlab.PlanetLabRunner


init(String) - Method in class org.cloudbus.cloudsim.examples.power.random.RandomRunner


init(String) - Method in class org.cloudbus.cloudsim.examples.power.RunnerAbstract
:   Inits the simulation.

init() - Static method in class org.workflowsim.failure.FailureGenerator
:   Initialize a Failure Generator.

init() - Static method in class org.workflowsim.failure.FailureMonitor
:   Initialize a FailureMonitor object.

init(Parameters.FTCMethod, Parameters.FTCMonitor, Parameters.FTCFailure, Map, int, String, String, String, OverheadParameters, ClusteringParameters, Parameters.SCHMethod, Parameters.PLNMethod, String, long) - Static method in class org.workflowsim.utils.Parameters
:   A static function so that you can specify them in any place

init(ReplicaCatalog.FileSystem) - Static method in class org.workflowsim.utils.ReplicaCatalog
:   Initialize a ReplicaCatalog

initialize() - Static method in class org.cloudbus.cloudsim.core.CloudSim
:   Initialise the simulation for stand alone simulations.

initialize(int) - Method in class org.cloudbus.cloudsim.network.FloydWarshall\_Float
:   initialization matrix

initLogOutput(boolean, boolean, String, String, String, String, String) - Method in class org.cloudbus.cloudsim.examples.power.RunnerAbstract
:   Inits the log output.

INQUIRY\_GLOBAL\_RC\_LIST - Static variable in class org.cloudbus.cloudsim.DataCloudTags
:   Denotes a list of Replica Catalogue (RC) entities that are listed in other regional Data GIS
    entities.

INQUIRY\_LOCAL\_RC\_LIST - Static variable in class org.cloudbus.cloudsim.DataCloudTags
:   Denotes a list of all Replica Catalogue (RC) entities that are listed in this regional Data
    GIS entity.

INQUIRY\_RC\_LIST - Static variable in class org.cloudbus.cloudsim.DataCloudTags
:   Denotes a list of Replica Catalogue IDs.

INQUIRY\_RC\_RESULT - Static variable in class org.cloudbus.cloudsim.DataCloudTags
:   Denotes a result regarding to a list of Replica Catalogue IDs.

INSIGNIFICANT - Static variable in class org.cloudbus.cloudsim.core.CloudSimTags
:   Denotes insignificant simulation entity or time.

iqr(double[]) - Static method in class org.cloudbus.cloudsim.util.MathUtil
:   Gets the IQR.

IqrMc - Class in org.cloudbus.cloudsim.examples.power.planetlab
:   A simulation of a heterogeneous power aware data center that applies the Inter Quartile Range
    (IQR) VM allocation policy and Maximum Correlation (MC) VM selection policy.

IqrMc() - Constructor for class org.cloudbus.cloudsim.examples.power.planetlab.IqrMc


IqrMc - Class in org.cloudbus.cloudsim.examples.power.random
:   A simulation of a heterogeneous power aware data center that applies the Inter Quartile Range
    (IQR) VM allocation policy and Maximum Correlation (MC) VM selection policy.

IqrMc() - Constructor for class org.cloudbus.cloudsim.examples.power.random.IqrMc


IqrMmt - Class in org.cloudbus.cloudsim.examples.power.planetlab
:   A simulation of a heterogeneous power aware data center that applies the Inter Quartile Range
    (IQR) VM allocation policy and Minimum Migration Time (MMT) VM selection policy.

IqrMmt() - Constructor for class org.cloudbus.cloudsim.examples.power.planetlab.IqrMmt


IqrMmt - Class in org.cloudbus.cloudsim.examples.power.random
:   A simulation of a heterogeneous power aware data center that applies the Inter Quartile Range
    (IQR) VM allocation policy and Minimum Migration Time (MMT) VM selection policy.

IqrMmt() - Constructor for class org.cloudbus.cloudsim.examples.power.random.IqrMmt


IqrMu - Class in org.cloudbus.cloudsim.examples.power.planetlab
:   A simulation of a heterogeneous power aware data center that applies the Inter Quartile Range
    (IQR) VM allocation policy and Minimum Utilization (MU) VM selection policy.

IqrMu() - Constructor for class org.cloudbus.cloudsim.examples.power.planetlab.IqrMu


IqrMu - Class in org.cloudbus.cloudsim.examples.power.random
:   A simulation of a heterogeneous power aware data center that applies the Inter Quartile Range
    (IQR) VM allocation policy and Minimum Utilization (MU) VM selection policy.

IqrMu() - Constructor for class org.cloudbus.cloudsim.examples.power.random.IqrMu


IqrRs - Class in org.cloudbus.cloudsim.examples.power.planetlab
:   A simulation of a heterogeneous power aware data center that applies the Inter Quartile Range
    (IQR) VM allocation policy and Random Selection (RS) VM selection policy.

IqrRs() - Constructor for class org.cloudbus.cloudsim.examples.power.planetlab.IqrRs


IqrRs - Class in org.cloudbus.cloudsim.examples.power.random
:   A simulation of a heterogeneous power aware data center that applies the Inter Quartile Range
    (IQR) VM allocation policy and Random Selection (RS) VM selection policy.

IqrRs() - Constructor for class org.cloudbus.cloudsim.examples.power.random.IqrRs


isActive() - Method in class org.cloudbus.cloudsim.HostStateHistoryEntry
:   Checks if is active.

isBeingInstantiated() - Method in class org.cloudbus.cloudsim.Vm
:   Checks if is being instantiated.

isDisabled() - Static method in class org.cloudbus.cloudsim.Log
:   Checks if the output is disabled.

isDisableMigrations() - Method in class org.cloudbus.cloudsim.power.PowerDatacenter
:   Checks if is disable migrations.

isEnableOutput() - Method in class org.cloudbus.cloudsim.examples.power.RunnerAbstract
:   Checks if is enable output.

isFailed() - Method in class org.cloudbus.cloudsim.Host
:   Checks if is failed.

isFinished() - Method in class org.cloudbus.cloudsim.Cloudlet
:   Checks whether this Cloudlet has finished execution or not.

isFinishedCloudlets() - Method in class org.cloudbus.cloudsim.CloudletScheduler
:   Informs about completion of some cloudlet in the VM managed by this scheduler.

isFinishedCloudlets() - Method in class org.cloudbus.cloudsim.CloudletSchedulerSpaceShared
:   Informs about completion of some cloudlet in the VM managed by this scheduler.

isFinishedCloudlets() - Method in class org.cloudbus.cloudsim.CloudletSchedulerTimeShared
:   Informs about completion of some cloudlet in the VM managed by this scheduler.

isFinishedCloudlets() - Method in class org.cloudbus.cloudsim.network.datacenter.NetworkCloudletSpaceSharedScheduler
:   Informs about completion of some cloudlet in the VM managed by this scheduler.

isFree() - Method in class org.cloudbus.cloudsim.network.datacenter.NetworkVm


isFull() - Method in class org.cloudbus.cloudsim.HarddriveStorage
:   Checks if the storage is full or not.

isFull() - Method in interface org.cloudbus.cloudsim.Storage
:   Checks if the storage is full or not.

isHostOverUtilized(PowerHost) - Method in class org.cloudbus.cloudsim.power.PowerVmAllocationPolicyMigrationAbstract
:   Checks if is host over utilized.

isHostOverUtilized(PowerHost) - Method in class org.cloudbus.cloudsim.power.PowerVmAllocationPolicyMigrationInterQuartileRange
:   Checks if is host over utilized.

isHostOverUtilized(PowerHost) - Method in class org.cloudbus.cloudsim.power.PowerVmAllocationPolicyMigrationLocalRegression
:   Checks if is host over utilized.

isHostOverUtilized(PowerHost) - Method in class org.cloudbus.cloudsim.power.PowerVmAllocationPolicyMigrationMedianAbsoluteDeviation
:   Checks if is host over utilized.

isHostOverUtilized(PowerHost) - Method in class org.cloudbus.cloudsim.power.PowerVmAllocationPolicyMigrationStaticThreshold
:   Checks if is host over utilized.

isHostOverUtilizedAfterAllocation(PowerHost, Vm) - Method in class org.cloudbus.cloudsim.power.PowerVmAllocationPolicyMigrationAbstract
:   Checks if is host over utilized after allocation.

isInMigration() - Method in class org.cloudbus.cloudsim.power.PowerDatacenter
:   Checks if PowerDatacenter is in migration.

isInMigration() - Method in class org.cloudbus.cloudsim.Vm
:   Checks if is in migration.

isInMigration() - Method in class org.cloudbus.cloudsim.VmStateHistoryEntry
:   Checks if is in migration.

isMasterCopy() - Method in class org.cloudbus.cloudsim.File
:   Checks whether this file is a master copy or replica

isMasterCopy() - Method in class org.cloudbus.cloudsim.FileAttribute
:   Checks whether this file is a master copy or replica

isNetworkEnabled() - Static method in class org.cloudbus.cloudsim.NetworkTopology
:   This method returns true if network simulation is working.

isPaused() - Static method in class org.cloudbus.cloudsim.core.CloudSim
:   Checks if is paused.

isReadOnly() - Method in class org.cloudbus.cloudsim.File
:   Checks whether this file is a read only or not

isReadOnly() - Method in class org.cloudbus.cloudsim.FileAttribute
:   Checks whether this file is a read only or not

isRegistered() - Method in class org.cloudbus.cloudsim.File
:   Checks if this file already registered to a Replica Catalogue

isRegistered() - Method in class org.cloudbus.cloudsim.FileAttribute
:   Checks if this file already registered to a Replica Catalogue

isSuitableForVm(Vm) - Method in class org.cloudbus.cloudsim.Host
:   Checks if is suitable for vm.

isSuitableForVm(Vm, long) - Method in class org.cloudbus.cloudsim.provisioners.BwProvisioner
:   Checks if BW is suitable for vm.

isSuitableForVm(Vm, long) - Method in class org.cloudbus.cloudsim.provisioners.BwProvisionerSimple


isSuitableForVm(Vm, int) - Method in class org.cloudbus.cloudsim.provisioners.RamProvisioner
:   Checks if is suitable for vm.

isSuitableForVm(Vm, int) - Method in class org.cloudbus.cloudsim.provisioners.RamProvisionerSimple


isWorking() - Method in class org.cloudbus.cloudsim.DatacenterCharacteristics
:   Checks whether all machines of this resource are working properly or not.

iteration - Static variable in class org.cloudbus.cloudsim.network.datacenter.NetworkConstants


iterator() - Method in class org.cloudbus.cloudsim.core.DeferredQueue
:   Returns an iterator to the events in the queue.

iterator() - Method in class org.cloudbus.cloudsim.core.FutureQueue
:   Returns an iterator to the queue.

A B C D E F G H I J L M N O P Q R S T U V W Z

- Overview
- Package
- Class
- Use
- Tree
- Deprecated
- Index
- Help

- Prev Letter
- Next Letter

- Frames
- No Frames

- All Classes
